# Human Data Science

**DOI:** 10.1016/j.patter.2020.100069

**Published:** 2020-07-10

**Authors:** DL Oberski

**Affiliations:** 1 Utrecht University and University Medical Center Utrecht, Ulrecht, the Netherlands

## Abstract

Most data science is about people, and opinions on the value of human data differ. The author offers a synthesis of overly optimistic and overly pessimistic views of human data science: it should become a science, with errors systematically studied and their effects mitigated—a goal that can only be achieved by bringing together expertise from a range of disciplines.

## Main Text

### “Data Are People!”

On the second of July, 1832, a 29-year-old data scientist named André-Michel Guerry presented a short manuscript at the prestigious *Académie Française*. His talk, published as *Essai sur la statistique morale de la France* (1833), changed the way that we view data, and it would also change the speaker’s own life, earning him an award and a coveted place in the academy.^1^ Up until that moment, Guerry had been an unassuming lawyer from a provincial town and had published works such as *Ancient folkloric chants of Poitou*, which he presumably wrote as a hobby to distract him from his initially dreary-seeming day job of compiling the French state’s crime data. During this time as a data manager, though, something marvelous appears to have occurred to Guerry: data on people were everywhere.

Readers in the era of personal computers, mobile phones, satellites, wearables, electronic health records, Google, business intelligence software, the internet, and the modern surveillance state will be as surprised by this insight as they are by tap water. But Guerry (along with his contemporary, Adolphe Quetelet) showed the world that we only need to reach out and analyze data that are already out there to learn more about the world and change our understanding of, for example, crime.^2^ In the time of Guerry and Quetelet, routinely produced and available data were overwhelmingly about people because they were produced for public administration by the state (hence “state-istics”). Our modern era is no different, in that the data leveraged in data science are predominantly collected in the course of public or private administration, and therefore about people.

In short, data science, since time immemorial, is mostly about humans: to mangle a quote from 1973 cult movie *Soylent Green*, “data is people!”

If data are people, how good are people? This appears to be a subject of some disagreement among philosophers, the comprehensive study of which is left as an exercise for the reader. For understanding of the key issues, here, the author will rely on TV. In the show *The Good Place* (NBC, 2016–2020), two opposing theses are proposed, which align closely with current data science practice. These are (1) everything is fine and (2) people are terrible. The purpose of this opinion piece is to combine these apparently contradictory theses into a new suggestion: that neither blind optimism nor blind despair are warranted, and data science must concentrate on developing the methodology to deal with the nature of humans and their data.

### Idea #1: Everything Is Fine

Data science has long been recognized as carrying great potential to improve people’s lives and gain insight. Some examples of applications currently at various stages of development and deployment include automatic segmentation of radiology images, processing of vast amounts of -omics data in bioinformatics, early warning on sepsis in neonatal intensive care units, prediction of housing market dynamics, or modeling of the spread of both infectious disease *and* anti-vaccination opinions through social networks.

#### A “Can-Do” Attitude

**Figure 1:**
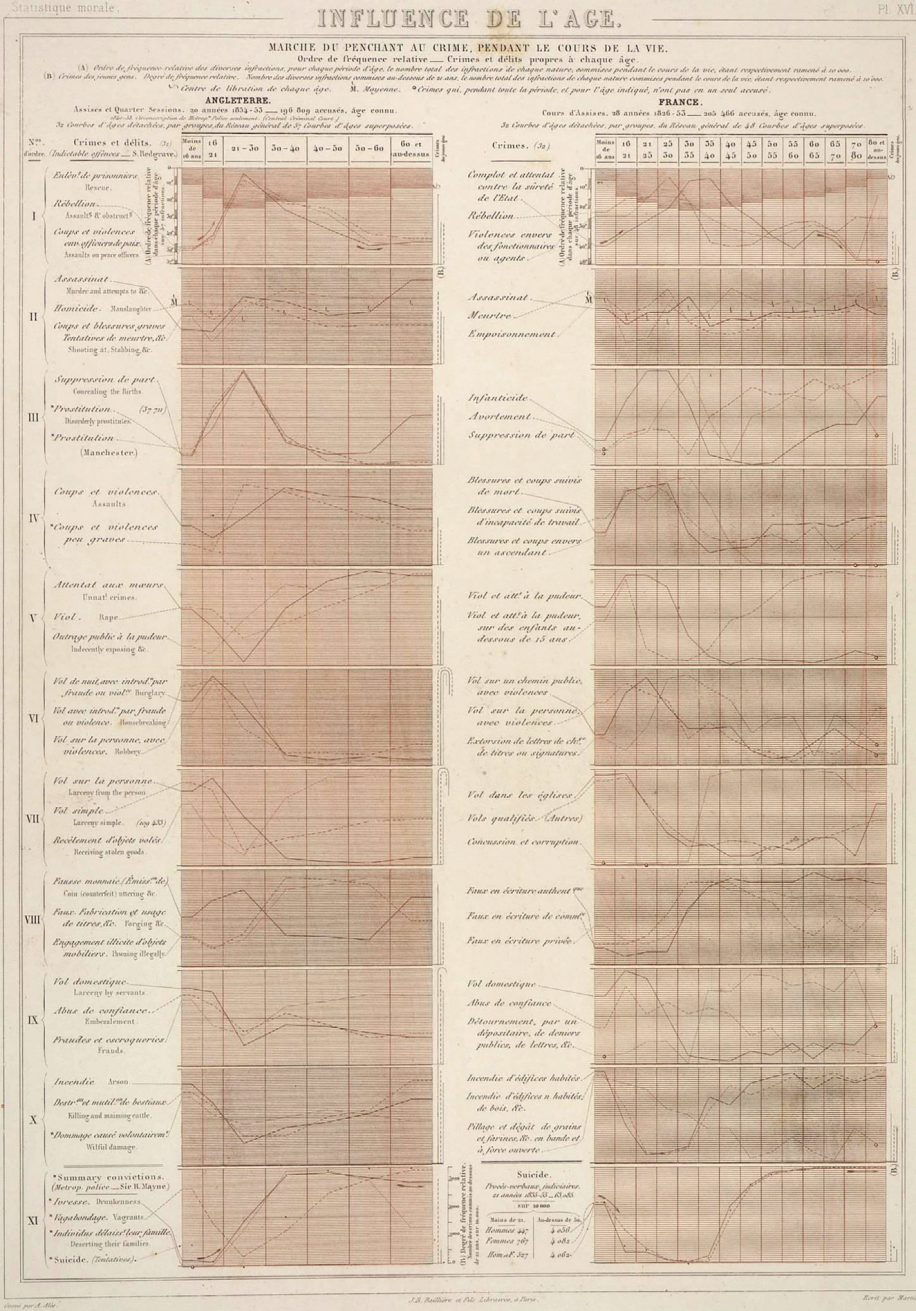
Guerry’s Visualization of Relative Crime Statistics across Age Groups from England and France Guerry’s visualization of relative crime statistics (vertical axes) across age groups (horizontal axes) from England (left-hand column) and France (right-hand column). Crimes with similar life-course patterns are grouped together. Courtesy of Universitat de Barcelona, Biblioteca Patrimonial Digital. License: Creative Commons Public Domain Mark 1.0.

A striking trait of many data scientists is a “can-do” attitude to data. For example, in making the beautiful graph reproduced in Figure 1, Guerry^1^ realized that England and France employed different definitions of crimes, that there were ample missing data and that the English data were less precise—note, for example, the differences in binning across the two columns (for a more detailed description of this and other visualizations, see Friendly^2^). But, thankfully, none of those issues appears to have plunged him into an impassable mental labyrinth of anxieties. Data science’s “can-do” attitude may have arisen from its partial roots in engineering, as engineers are used to making do; if MacGyver had spent his time philosophizing about the adhesive properties of duct-tape, he never would have escaped that shark tank. This positive spirit plays an important role in data science’s current popularity: it is exciting to think that we can understand more and make better decisions by purely using “found data” from all types of human activity. When Guerry presented his findings to the *Académie*, many of its illustrious members must have been pleasantly surprised that all the human data lying around could produce such interesting results.

#### Data Science Improves Continually

**Figure 2:**
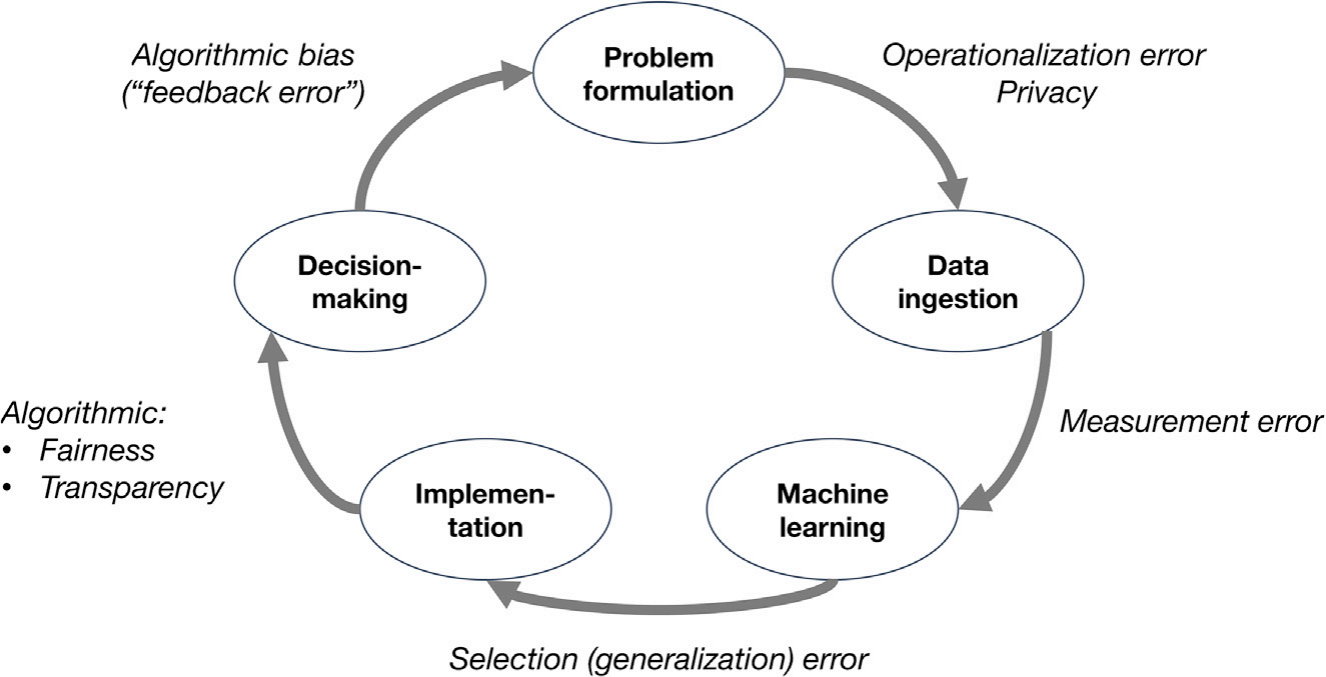
At Each Step of the Typical Human Data Science Cycle, Errors Occur

The inner circle in Figure 2 shows an approach to (human) data science in an idealized cycle of investigation and “product” building (where a “product” can also be an insight). I will briefly illustrate. Ideally, one would start with the *problem formulation*. For example, to reduce the costs and improve the benefits of treatment for a new infectious disease, one would design a system that will screen potential patients more accurately than is done currently. People that do not need treatment will receive it less often, reducing costs; patients that do need treatment can be recognized earlier, improving survival. We then need *data*. The ideal data, perfectly measured and perfectly randomized, are not available. But we make do with a large amount of data from an app in which people register their symptoms, as well as from the routine care processes of general practitioners (GPs) and hospitals, in which we find survival data and background variables. The next task is to assess how survival might be *predicted* from symptoms and background variables, so that we can screen people (“machine learning” is a commonly used term for this step, although some readers might prefer some other term, such as “prediction modeling”). Once that is done, and evaluated to everyone’s satisfaction, it is time to *implement* the screening, perhaps by changing the warning system in the app, or by developing a software plug-in to the GP’s electronic health record. And after the system has been implemented, it will influence actual *decision-making*, for example by guiding the decision to refer a patient to further care or advising home confinement. We can then keep observing and improving the system.

What could possibly go wrong?

### Idea #2: People Are Terrible

Among the various objects that fall within the purview of statistics, one of the most important and the most difficult to deal with, consists in the enumeration and classification of human actions…*Lacroix on A-M. Guerry’s* Essai sur la statistique morale de la France.^1^

”Pobody’s nerfect.”— Eleanor Shellstrop (*The Good Place*)

When dealing with human data, the can-do “everything is fine” is one idea. Its antithesis, embodied in TV’s *The Good Place* by the evil demon Shawn, is that humans are the worst. Specifically, they are hard to predict, they differ, they have rights, and they are like ourselves.

#### Humans Are Just Hard to Predict

We do not currently understand the health and societies of humans—or other complex organisms for that matter—the way we understand, say, bridgebuilding. Two centuries of quantitative social science have shown clear patterns in averages such as those shown in Figure 1. But, even using more data and more sophisticated models, it can be surprisingly difficult to improve upon existing, simplistic models for individual human health and behavior. For example, Christodoulou et al.’s systematic review^3^ of machine learning approaches to clinical prediction concluded “no performance benefit” of modern machine learning approaches over a simple benchmark; following the illustration, we may doubt whether our elaborate cycle will improve on a common-sense rule that simply says people who look sick should see a doctor. In sociology, Salganik et al.^4^ reported on the Fragile Families Challenge, a mass collaboration effort to measure the predictability of life outcomes such as layoff, GPA, or material hardship, that “despite using a rich dataset and applying machine-learning methods optimized for prediction, the best predictions were not very accurate and were only slightly better than those from a simple benchmark model.”

#### Humans Differ

“How are you feeling”? An innocuous question used in polite conversation, in social surveys, and at the doctor’s office. But, as doctors know, no two people answer this question in the same way. They might give the same *answer* but that does not mean they feel the same. Or they might give *different* answers but feel similar (a technical doctor-term for this is “crybaby”). This becomes a measurement error problem when self-reported symptoms are used to predict survival, which leads to problems when symptoms are used as ground-truth in an machine learning model. And before you start thinking self-reports are worse than so-called “objective data” from administrative records, wearables, apps, and the like, those data have huge errors as well (see Oberski et al.^5^ for references). Selection error is a problem too. For example, looking only at western, educated, industrialized, rich, and democratic (WEIRD) societies gives experimental results in behavioral science that are probably atypical for humans in general. In our illustration, not everyone uses an app. That is fine as long as future app users are similar to current app users, but, presumably, by improving the app more people will want to use it. So, we are changing the population. Because populations can shift over time, selection errors may lead to generalization problems.

#### Humans Have Rights

“‘Fair’ is the stupidest word humans ever invented, except for ‘staycation’.”—Shawn (*The Good Place*)

Common decency, and—for the demons among my readers—many laws, require that data science, when applied to humans, makes decisions that are fair and that do not have harmful side-effects, for example, through confidentiality breaches. In many cases, some degree of explainability or transparency of the decisions is also necessary. This means that prediction performance or the rigor of the scientific approach is not the only parameter to be optimized: instead, the entire cycle must be enacted under constraints, which can be considerable and which can have dire consequences when violated (see Obermeyer et al.^6^ for an example).

#### Humans “R” Us

Finally, dear reader, you and I are not the only smart ones. Our data subjects and product users are smart like us. When we advise them at the end of Figure 2’s “inner” data science cycle and try to go back to the beginning, they will think about our advice and either (1) ignore it or (2) not ignore it. Both can be bad. When they ignore us, the potential value we created with our data science cycle is wasted. But things can get even worse when people actually listen to us: people might trust the results too much, for example, making decisions that do not properly account for the associated uncertainties, and leading to potentially large losses. In our illustration, patients kept at home by a human doctor might be followed up more than patients being kept at home by an all-knowing computer, for instance. Another problem is that of algorithmic feedback. To illustrate, suppose that, in our data, people with a fever survive equally well compared with other symptomatic patients, so that this variable is not used to predict survival. But perhaps this was because the feverish go to the hospital early and therefore get better treatment, canceling out their otherwise bleaker prospects. The absence of an observed association has erroneously convinced our model that people with fever have nothing to worry about. After implementing the model, this (1) worsens their survival and (2) decreases model accuracy (I leave it up to the reader’s conscience to decide which is the more pressing reason for caution).

In short, the second thesis is: sure, it seems attractive to enter the inner circle in Figure 2. But at each step of the process, in the outer circle of Figure 2, demons stand ready with the pitchforks of measurement and selection error, fairness, transparency, privacy risks, and algorithmic bias to torment us. All who enter here, despair!

### A Better Idea: Let’s Science Our Problems

“In football, trying to run out the clock and hoping for the best never works. It’s called ‘prevent defense.’ You don’t take any chances and just try and hang on to your lead. But prevent defense just *prevents* you from winning. It’s always better to try something”—Jason Mendoza (*The Good Place*)

In summary, an, admittedly caricatured, picture of the current situation is this: we either assume everything is fine (idea #1), or we dismiss human data out of hand (idea #2).

Because neither is particularly constructive, we should do neither. Instead, we should concentrate on the *science of human data science*. Good human data science:

(1) Acknowledges that errors—in data, modeling decisions, and implementation—are inevitable;
(2) Studies the degree to which errors actually affect the output;
(3) Removes these effects where necessary, either by preventing the errors or, when this is not possible or cost-effective, by correcting for their effects.

In keeping with data science’s engineering roots, good human data science is also good engineering. A good engineer does not just plow ahead, regardless of the terrain. Instead, she designs a solution that uses the available materials, while controlling the risks. These risks have been recognized from the very beginning for human data science; even the person who wrote Guerry’s introduction called human data the “most difficult to handle.” And he was probably being polite.

Here are some potential things one could do to mitigate risks by approaching human data science as a science:

• Work closely with domain experts to formulate a useful problem and operationalize it into a data science project appropriately;
• Work closely with domain experts to understand the evidence that including certain variables (features) will yield a net benefit;
• Don’t assume measurement error will “cancel out.” Use statistical models to estimate and correct for the effect of measurement errors;
• Use causal models and research designs to estimate and correct for the effects of selection error, including missing data;
• Investigate any threats to fairness of decision-making, and use appropriate techniques to ensure models do not learn biases;
• Use appropriate techniques to explain the model results to users and data subjects;
• Work with cognitive scientists to evaluate whether the above actually worked;
• Work with social scientists and domain experts to investigate how the data product affects daily practice.

#### How Do We Work toward Achieving These Lofty Goals?

First, *latent variable models and causal modeling can provide a convenient framework* to think about some of these issues. For example, latent variable models are a convenient framework to estimate and correct for data issues,^5^ causal modeling is a useful tool in dealing with selection error,^7^ and both causal modeling and latent variables are useful tools when investigating model output’s fairness (see Boeschoten et al.^8^ and references therein).

Second, human data scientists need to routinely take a much wider view of “model evaluation”; a successful data science project must not only predict well in out-of-sample data, but also generalize to other contexts in which it might be applied, generalize to the concept it was intended to study, reflect uncertainty accurately, improve on what was already there, have benefits that outweigh its costs, and actually achieve its stated goals in social reality. To verify all of these, the *comprehensive scientific study of data science projects should become routine*.

Third, we should collaborate much more. I do not pretend to be the first person to point out these issues are important; many of the points above are already entire research fields, covering swathes of statistics, computer science, ethical and legal scholarship, domain sciences such as medicine and biology, and social science. But, in my opinion, we are not collaborating enough, and we are not bringing in a large enough diversity of perspectives. In going through the data science cycle, it is all too easy to focus on what interests us, to the detriment of the project as a whole. No one person, including your author, is immune to this problem! For example, some research in data science ethics focuses exclusively on ethical, legal, and societal aspects and pass over existing mathematical tooling to weigh the issues in a practical engineering context. Conversely, some other research focuses entirely on the mathematical tooling without considering whether it will be of practical use in a social context: have I done justice to people’s intelligence and autonomy; will people actually react the way I assume they will? *Of course* no one person can do everything. That is exactly why we should work harder to unite *all* of the fields that work on these problems.

I am aware both that my to-do list for the science of human data science is long and that it should be longer. But, if we really want to put human data to good use, it is the work we need to do. The demons are legion, and nobody ever said it was going to be easy to avoid them. But, as André-Michel Guerry, Jason Mendoza, and many others have shown, it will be well worth it. So, let’s science the heck out of human data science!
